# Optimal proteome allocation and the temperature dependence of microbial growth laws

**DOI:** 10.1038/s41540-021-00172-y

**Published:** 2021-03-08

**Authors:** Francis Mairet, Jean-Luc Gouzé, Hidde de Jong

**Affiliations:** 1 Ifremer, Physiology and Biotechnology of Algae laboratory, Nantes, France; 2 Université Côte d’Azur, Inria, INRAE, CNRS, Sorbonne Université, Biocore team, Sophia Antipolis, France; 3 Université Grenoble Alpes, Inria, Grenoble, France

**Keywords:** Systems analysis, Microbiology, Differential equations

## Abstract

Although the effect of temperature on microbial growth has been widely studied, the role of proteome allocation in bringing about temperature-induced changes remains elusive. To tackle this problem, we propose a coarse-grained model of microbial growth, including the processes of temperature-sensitive protein unfolding and chaperone-assisted (re)folding. We determine the proteome sector allocation that maximizes balanced growth rate as a function of nutrient limitation and temperature. Calibrated with quantitative proteomic data for *Escherichia coli*, the model allows us to clarify general principles of temperature-dependent proteome allocation and formulate generalized growth laws. The same activation energy for metabolic enzymes and ribosomes leads to an Arrhenius increase in growth rate at constant proteome composition over a large range of temperatures, whereas at extreme temperatures resources are diverted away from growth to chaperone-mediated stress responses. Our approach points at risks and possible remedies for the use of ribosome content to characterize complex ecosystems with temperature variation.

## Introduction

Predicting microbial growth rate is of significant interest, from the comprehension of ecosystems to the optimization of biotechnological processes^1^. A fruitful perspective on microbial growth is provided by the study of resource allocation. Assuming that microorganisms have acquired through evolution strategies for maximizing their fitness, metabolism can be represented as an optimization problem, where resources (e.g., proteins) must be affected to different sectors—substrate uptake, ATP production, protein synthesis, etc. - so as to maximize growth rate or energy efficiency^2–5^. This framework has resulted in microbial growth laws in agreement with experimental observations, such as the linear relationship between growth rate and ribosome content during balanced growth^6–10^. Most of this work, however, has focused on the effect of nutrient limitations. How the growth laws are affected by other environmental factors or stresses remains an open question.

One key factor is temperature, and its impact on microbial growth has been widely studied. Above all, an increase in temperature speeds up reaction kinetics, but it also has an effect on, for example, protein stability and membrane fluidity^1,11,12^. Heat stress causes the accumulation of misfolded proteins which can perturb cellular functions^13^, besides representing a loss of resources available for growth. Under such stressful conditions, many microorganisms produce chaperones to support (re)folding^14^, and proteases to degrade denatured proteins^15^. Since its discovery in the seventies, this so-called heat shock response has been well characterized^16–18^. Similarly, protein denaturation and chaperone production have also been observed under cold shock^19,20^.

Despite these studies, it remains unclear how global resource allocation underlies the effect of temperature on cellular physiology and growth. Recently, this question has been tackled using a genome-scale protein-folding model, called FoldME^21^. The linear relationship between growth rate and ribosome content—well-established when the growth rate is varied by supplying different substrates^9^ - is obtained in silico for different temperatures as well (see Fig. 3A in Chen et al.^21^). However, Herendeen et al.^22^ have shown that over a large (20 K) temperature range the ribosomal content remains relatively constant, whereas the specific growth rate increases with temperature. Clarifying this discrepancy between model and experiments is not only interesting for gaining a better understanding of the relationship between temperature and growth, but is also of high interest for ecology. In this field, the ribosome (or RNA) content has been used to estimate growth rate or, more generally, microbial activity. Several studies, however, have cast serious doubts on the overall reliability of the method^23^, which may be due, among other things, to the interference of environmental factors like temperature variations^24^.

In this context, our objective is to provide quantitative insight into the way resource allocation constraints shape the effect of temperature on growth, and compare it with the effect of substrate limitation. In particular, how does temperature affect ribosome and chaperone contents? Can we predict proteome sector allocation based on an appropriate optimization principle? Is there a generalization of the microbial growth law that accounts for temperature effects? To answer these questions, we use a coarse-grained model of microbial growth, including two macroreactions for precursor and protein synthesis, and the processes of temperature-sensitive protein unfolding and chaperone-assisted folding. We determine optimal resource allocation during balanced growth as a function of substrate limitation and temperature. After its calibration with quantitative proteomic data for *Escherichia coli*^25^ and two thermal growth curves^26,27^, the model is used to bring out general principles of proteome allocation, supported by experimental data. In particular, the model correctly predicts that the proteome composition remains relatively constant over a 20 K range, while the growth rate increases with temperature due to the overall increase of reaction rates. The model shows that this observation can be accounted for by the approximately equal activation energies for metabolic enzymes and ribosomes. Moreover, optimization arguments also explain that, at extreme temperatures, the chaperone content increases to cope with protein unfolding, at the expense of metabolic and ribosomal proteins. Finally, based on these insights, we derive microbial growth laws corrected for temperature effects. This generalization can notably help to improve the exploitation of measurements of ribosome (or RNA) content in ecology for characterizing the functioning of complex ecosystems.

## Results

### Modeling microbial resource allocation

**Fig. 1:**
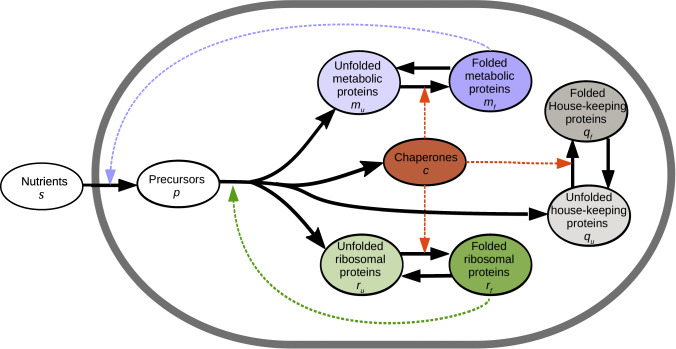
Outline of the coarse-grained model. Proteins are divided into four main sectors: metabolic proteins (at mass fraction *m*, in units g/g prot) which convert substrate (*s*) into precursors (*p*), ribosomal proteins (*r*) which synthetize proteins, chaperones (*c*) which fold proteins, and house-keeping proteins (*q*). Within the protein sectors, apart from the chaperones, we distinguish between folded and unfolded proteins, as indicated by the indices *f* and *u*, respectively. Solid arrows refer to mass flows and dashed arrows to catalytic (enzymatic) activities.

Our coarse-grained model represents microbial growth as a resource allocation problem under the effect of nutrient limitation and temperature, extending the model proposed in Giordano et al.^5^. Except for the external substrate, all variables represent mass fractions (expressed in gram per gram of proteins, assuming that the mass of precursors is negligible compared to that of proteins). Growth is represented by two macroreactions (Fig. 1). First, substrate at concentration *s* is converted into precursors (mainly amino acids) at mass fraction *p*. These precursors are then used to produce proteins, divided into four sectors: chaperones at mass fraction *c*, metabolic proteins (*m*), active ribosomal proteins (*r*), and house-keeping proteins (*q*) (the latter includes a minimum reserve of inactive ribosomes *r*_0_^28,29^). The allocation variables *α*_*c*_, *α*_*m*_, *α*_*r*_, and *α*_*q*_ describe how the protein synthesis flux is distributed over the four sectors. By definition, these allocation variables are positive and satisfy *α*_*c*_ + *α*_*m*_ + *α*_*r*_ + *α*_*q*_ = 1. Metabolic, ribosomal, and house-keeping proteins are either folded or unfolded (denoted, respectively, with subscripts *f* and *u*, e.g., *m* = *m*_*f*_ + *m*_*u*_).

The kinetics of precursor and protein synthesis *v*_*M*_ and *v*_*R*_ are represented by Michaelis-Menten functions, catalyzed by (folded) metabolic and ribosomal proteins, respectively:

1 $${v}_{M}(s,{m}_{f})={k}_{M}\frac{s}{{K}_{s}+s}{m}_{f},\ \ {\rm{and}}\ \ {v}_{R}(p,{r}_{f})={k}_{R}\frac{p}{{K}_{p}+p}{r}_{f},$$

where *k*_*M*_, *k*_*R*_ are maximal rates (in /h), and *K*_*s*_, *K*_*p*_ half-saturation constants (in g/L and g/g prot, respectively). In the following, the fractional term in *v*_*M*_ will be denoted *g*(*s*) = *s*/(*K*_*s*_ + *s*).

In line with El-Samad et al.^17^, mass-action kinetics are used for chaperone-assisted folding:

$${v}_{f}^{m}({m}_{u},c)={k}_{f}{m}_{u}c,\ \ {v}_{f}^{r}({r}_{u},c)={k}_{f}{r}_{u}c,\ \ {v}_{f}^{q}({q}_{u},c)={k}_{f}{q}_{u}c,$$

and unfolding:

$${v}_{u}^{m}({m}_{f})={k}_{u}{m}_{f},\ \ {v}_{u}^{r}({r}_{f})={k}_{u}{r}_{f},\ \ {v}_{u}^{q}({q}_{f})={k}_{u}{q}_{f}$$

of metabolic, ribosomal, and house-keeping proteins, respectively. In the absence of precise information, we take the same kinetic parameters *k*_*f*_ and *k*_*u*_ for the three protein sectors.

On the time-scale of interest, i.e., the time-scale of the dynamics of the total protein mass fractions *m*, *r*, and *q*, the folding processes are fast (with a characteristic time of 0.1 s, compared to 10 s for protein synthesis^30^). Using slow-fast approximations^31^, the dynamics of folding-unfolding are assumed to converge towards their quasi-steady state given by ${v}_{f}^{m}({m}_{u},c)={v}_{u}^{m}({m}_{f})$, ${v}_{f}^{r}({r}_{u},c)={v}_{u}^{r}({r}_{f})$, and ${v}_{f}^{q}({q}_{u},c)={v}_{u}^{q}({q}_{f})$, which leads to:

2 $${m}_{f}=\frac{{k}_{f}c}{{k}_{u}+{k}_{f}c}m,\ {r}_{f}=\frac{{k}_{f}c}{{k}_{u}+{k}_{f}c}r,\ {q}_{f}=\frac{{k}_{f}c}{{k}_{u}+{k}_{f}c}q.$$

The effect of temperature *T* (in K) is represented by Arrhenius functions for all reaction kinetics, except for protein unfolding which is more sensitive to temperature^17^. Based on a reference temperature *T*_*ref*_, we express the rates as follows:

3 $${k}_{M}={k}_{M}^{ref}{\varphi }_{M}(T),\ {k}_{R}={k}_{R}^{ref}{\varphi }_{R}(T),\ {k}_{f}={k}_{f}^{ref}{\varphi }_{f}(T),\ {k}_{u}={k}_{u}^{ref}\psi (T).$$

The Arrhenius functions *φ*_*M*_(*T*), *φ*_*R*_(*T*), and *φ*_*f*_(*T*) are characterized by their activation energies (in J/mol) *E*_*M*_, *E*_*R*_, and *E*_*f*_, e.g.,

$${\varphi }_{M}(T)=\exp \left(\frac{-{E}_{M}}{R}\left(\frac{1}{T}-\frac{1}{{T}_{ref}}\right)\right),$$

where *R* is the universal gas constant (in J/(mol ⋅ K)). Deviations from the Arrhenius law have been observed for protein unfolding^32–34^, so a phenomenological equation *ψ*(*T*) is chosen:

$$\psi (T)={\exp }_{d}\left(\frac{-{E}_{u}}{R}\left(\frac{1}{T}-\frac{1}{{T}_{ref}}\right)\right),$$

where ${\exp }_{d}$ is the deformed exponential function given by ${\exp }_{d}(x)\equiv {(1+dx)}^{\frac{1}{d}}$^35^, *E*_*u*_ is the pseudo activation energy and *d* is the deformation parameter (the usual exponential function is recovered when *d* → 0). *φ* and *ψ*, plotted in Supplementary Fig. 4B, are both increasing convex functions of T.

By adding these features, we obtain from the mass balances a dynamical model for microbial growth (see Supplementary Material for more details on model derivation):

4 $$\left\{\begin{array}{l}\frac{dp}{dt}\,\,=\,\,{v}_{M}(s,{m}_{f})-(1+p){v}_{R}(p,{r}_{f}),\\ \frac{dc}{dt}\,\,=\,\,({\alpha }_{c}-c){v}_{R}(p,{r}_{f}),\\ \frac{dm}{dt}\,=\,({\alpha }_{m}-m){v}_{R}(p,{r}_{f}),\\ \frac{dr}{dt}\,\,=\,\,({\alpha }_{r}-r){v}_{R}(p,{r}_{f}),\\ \frac{dq}{dt}\,=\,({\alpha }_{q}-q){v}_{R}(p,{r}_{f}),\end{array}\right.$$

with *m*_*f*_ and *r*_*f*_ given by Eq. (2), and the kinetics given by Eqs. (1) and (3). Finally, assuming that the volume of the growing population is proportional to protein mass, the specific growth rate *μ* is given by the protein synthesis rate:

$$\mu ={v}_{R}(p,{r}_{f}).$$

This coarse-grained model—based on mechanistic assumptions—allows us to represent how the proteome allocation variables (*α*_*c*_, *α*_*m*_, *α*_*r*_, *α*_*q*_) affect the microbial growth rate and cellular composition, including the effects of temperature and substrate limitation.

### Determining optimal resource allocation in balanced growth

A commonly-made assumption is that microorganisms have evolved so as to optimize their growth rate^2,3^, so our objective is to find (*α*_*c*_, *α*_*m*_, *α*_*r*_) maximizing *μ* in balanced growth conditions. In line with Scott at el.^3^, we consider the system in a constant environment at steady state, representing balanced growth conditions, and we assume that the house-keeping allocation *α*_*q*_ is fixed (and therefore *q* as well, which equals *α*_*q*_ at steady-state). Formally, this gives an optimization problem under constraints:

5 $$\begin{array}{ll}({\alpha }_{c}^{opt},{\alpha }_{m}^{opt},{\alpha }_{r}^{opt})=&\mathop{{\mathrm{arg}}\,{\mathrm{max}}}\limits_{({\alpha }_{c},{\alpha }_{m},{\alpha }_{r})\in {[0,1]}^{3}}\mu \\ &{\rm{such}}\ {\rm{that}}\ \left|\begin{array}{l}\frac{dp}{dt}=\frac{dc}{dt}=\frac{dm}{dt}=\frac{dr}{dt}=\frac{dq}{dt}=0,\\ {\alpha }_{c}+{\alpha }_{m}+{\alpha }_{r}+{\alpha }_{q}=1.\end{array}\right.\end{array}$$

The optimal solution can be determined analytically (see Method for details):

6 $$\left\{\begin{array}{l}{p}^{opt}\,=\,\sqrt{\frac{{k}_{M}g(s){K}_{p}}{{k}_{R}}}\,,\\ {\alpha }_{c}^{opt}\,=\,{c}^{opt}\ =\ \frac{{k}_{u}}{{k}_{f}}\left(-1+\sqrt{1+(1-q)\frac{{k}_{f}}{{k}_{u}}}\right),\\ {\alpha }_{r}^{opt}\,=\,{r}^{opt}\ =\ (1-q-{c}^{opt})\frac{{p}^{opt}({K}_{p}+{p}^{opt})}{{K}_{p}+2{K}_{p}{p}^{opt}+{{p}^{opt}}^{2}}\ ,\\ {\alpha }_{m}^{opt}\,=\,{m}^{opt}\ =\ (1-q-{c}^{opt})\frac{{K}_{p}(1+{p}^{opt})}{{K}_{p}+2{K}_{p}{p}^{opt}+{{p}^{opt}}^{2}}\ .\end{array}\right.$$

Before comparing this result with experimental data, a structural property of the above equations gives a useful biological insight. First, by analyzing the second equation of (6), one can check that the optimal chaperone content increases with the ratio *k*_*u*_/*k*_*f*_, i.e., when unfolding becomes more prominent in comparison with folding. In addition, for any positive value of *k*_*u*_/*k*_*f*_, we have:

$$0\,<\,{c}^{opt}\,<\,\frac{1}{2}(1-q).$$

As a consequence, considering the two last equations of (6), we can verify that (*m*^*opt*^, *r*^*opt*^) ∈ [0, 1]^2^, and that

$$\frac{1}{2}(1-q)\,<\,{r}^{opt}+{m}^{opt}\,<\,(1-q).$$

That is, in our model, the optimal chaperone mass fraction is always lower than the sum of the metabolic and ribosomal protein sectors. This is confirmed by experimental data: in *E. coli*, the two major chaperone systems represent 15–20% of total protein at 46 ^∘^C ^16^, while the metabolic and ribosomal protein sectors sum to 35–40%, assuming that the house-keeping protein sector amounts to 45%^9^.

### The model reproduces thermal growth response and resource allocation under substrate limitation

**Fig. 2:**
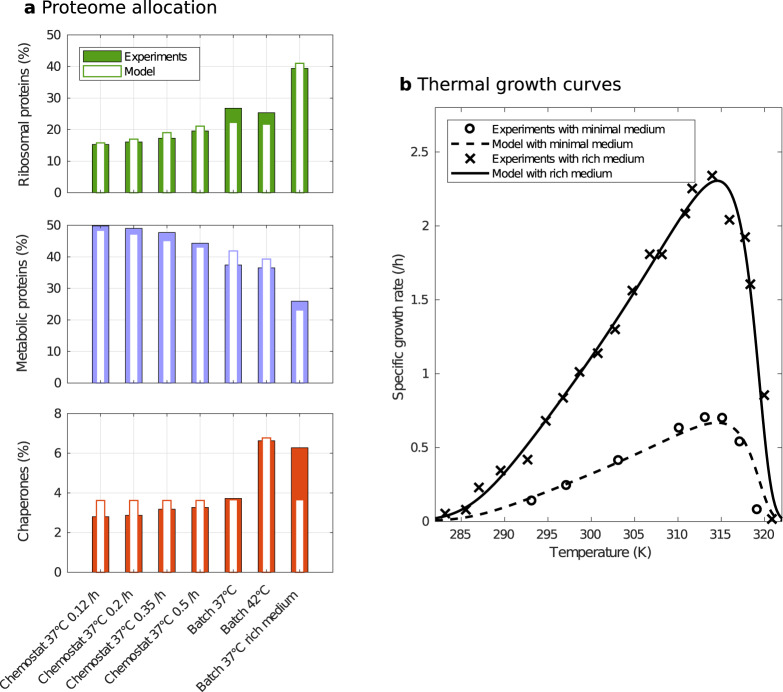
Calibration of the coarse-grained resource allocation model with experimental data for *E. coli*. **a** Proteome allocation to different sectors in % of total protein mass for seven conditions, namely growth in chemostat at 37 ^o^C (dilution rates of 0.12/h, 0.2/h, 0.35/h, and 0.5/h), and in batch (exponential phase) at 37 ^o^C and 42 ^o^C in glucose minimal medium, and at 37 ^o^C in rich medium (data from ref. ^25^). **b** Specific growth rate as a function of temperature in glucose minimal and rich media (data from refs. ^26^^,^^27^, respectively).

We first test to which extent the optimal solution (6) can reproduce available experimental data. To this end, we fit the model parameters using experimental data from *E. coli*, namely two thermal growth response curves^26,27^ and proteomic data for seven conditions^25^, starting from initial parameter guesses from the literature. Alternative fits have been carried out to test if it is necessary to consider different activation energies for the Arrhenius functions (see Method for more details on parameter estimation). The best fit in terms of the Akaike information criterion (AIC) is obtained when considering the same activation energies for precursor and protein synthesis (i.e., *E*_*M*_ = *E*_*R*_ ≠ *E*_*f*_). Figure 2 shows that the optimal solution fits the data well for both the proteome allocation profiles and the growth rates, with a median relative error of 7.9% and 4.2%, respectively. This suggests that the assumptions underlying our model are capable of quantitatively accounting for the observations. In particular, the model captures the asymmetry of the thermal response^1^: the growth rate increases exponentially with temperature until a maximum, and then suddenly drops. The parameter values obtained from model calibration (given in Table 2) are almost all of the same order of magnitude as the reference values, with a mean log10 fold-change of 0.49 (a log10 fold-change of 1 corresponds to a difference of one order of magnitude). This further supports model soundness.

### The model qualitatively predicts effect of temperature on resource allocation

**Fig. 3:**
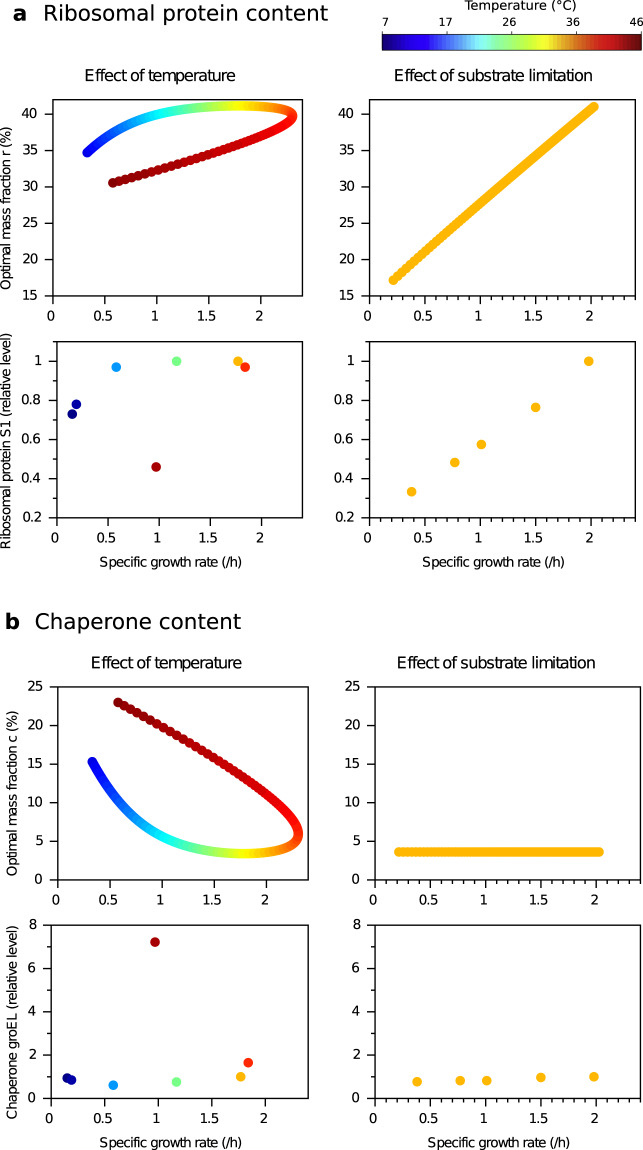
Protein sector content for ribosomal proteins and chaperones as a function of specific growth rate, varying with temperature (left) or substrate limitation (right). **a** Ribosomal protein mass fraction. Top: model prediction. Down: Level of the ribosomal protein S1 in *E. coli* for different temperatures in glucose rich medium (left), and for different media at 37 ^∘^C, relative to glucose-rich medium at 37 ^∘^C^22,36^. For each point, color represents temperature (see colorbar). **b** Chaperone mass fraction. Same legend as (**a**) for the chaperone GroEL.

The model can be used to understand how temperature affects proteome allocation in balanced growth conditions. To do so, we predict the optimal resource allocation profile as a function of growth rate for different temperatures or substrate limitations, and compare qualitatively these predictions with experimental data (not used for calibration) for *E. coli*^22,36^. The results obtained with the parameter set given in Table 2 (with *E*_*M*_ = *E*_*R*_) are presented in Fig. 3 (see also Supplementary Fig. 5A for model prediction with alternative fits). The model predicts that the ribosomal and chaperone sectors are almost constant over a 20 K temperature range. But at lower or higher temperatures, the ribosomal sector decreases, whereas the chaperone sector increases. By comparison, under substrate limitation, the ribosomal sector is predicted to increase with growth rate—reproducing a well-known growth law ^9^—while chaperones remain constant (this is expected from the analytical solution, in which the optimal chaperone content depends on temperature but not on substrate concentration).

The predicted resource allocation profiles correspond remarkably well with the experimental data, for all the identified chaperones and ribosomal proteins present in refs. ^22,36^, as shown in Fig. 3 and in Supplementary Figs. 1 and 2. In particular, at non-extreme temperatures, proteins remain mostly folded and the metabolic and translational rates follow the same Arrhenius increase. Thus, no reallocation is needed: proteome allocation remains constant, while the growth rate increases in accordance with the temperature-dependent increase in reaction rates. Actually, Farewell and Neidhardt^37^ already observed for *E. coli* that the specific growth rate and the translation elongation rate increase similarly with temperature, up to 37 ^∘^C. On the other hand, at extreme temperatures, the model captures how a stress diverts resources away from growth. Low or high temperatures favor protein unfolding, and eventually call for the production of chaperones to counteract these deleterious effects, at the expense of ribosomal and metabolic proteins.

Finally, one may wonder if what is true for *E. coli* also holds for other species. The RNA content (often used as a proxy for ribosomal proteins) of the yeast *Saccharomyces cerevisiae*^24,38^ for different temperatures and levels of substrate limitation shows the same trends (see Supplementary Fig. 3). This suggests the generality of the model beyond enterobacteria.

### Correction of the microbial growth law for temperature effects

The correlation between the ribosome content and the specific growth rate—observed over different levels of substrate limitation at the same temperature—is a well-known microbial growth law^9^. However, this dependence changes completely when the growth rate varies due to the temperature, as we have seen in the previous section: the growth rate increases with temperature, while the ribosome content remains the same over a rather wide range of temperatures. Using the resource allocation model, our objective here is to generalize the growth law in order to take into account the above temperature effects.

Using the expression of the optimal allocation (6), we get a linear relationship between the specific growth rate *μ* and the total ribosome content *r*_*tot*_ (obtained by summing the active ribosome content *r* and the minimum reserve of inactive ribosomes *r*_0_) for a constant temperature (see Method for details):

7 $${r}_{tot}=\frac{1}{{k}_{R}^{ref}\eta (T){\varphi }_{R}(T)}\mu +{r}_{0},$$

where *η* is the ratio of folded over total proteins, which depends only on temperature:

8 $$\eta (T)=1-{\left[1+(1-q)\frac{{k}_{f}(T)}{{k}_{u}(T)}\right]}^{-1/2}.$$

Importantly, the slope of the linear relationship (7) (i.e., the classical growth law^9^) depends on temperature, and more precisely on the product of *η*(*T*) and *φ*_*R*_(*T*). This reflects the distinct effects of temperature on protein stability (as reflected by the folded protein ratio *η*) and activity (*φ*_*R*_). As explained above, both effects influence the relation between growth rate and ribosome content. Note that the optimal allocation leads to a constant (and maximum) ribosome efficiency due to precursor saturation, in line with experimental observations^39,40^. Consequently, the growth rate depends only on the ribosome content (and not on the precursor concentration).

To consider the temperature effect on reaction rates, we define the Arrhenius-corrected growth rate *ν*:

9 $$\nu =\frac{\mu }{{\varphi }_{R}(T)}=\mu \exp \left(\frac{{E}_{R}}{R}\left(\frac{1}{T}-\frac{1}{{T}_{ref}}\right)\right).$$

This generalized definition is in agreement with the temperature correction introduced in the metabolic theory of ecology^41^, which aims at providing an integrated view of energy and material fluxes, from individual organisms to ecosystems, including the effect of temperature (given that the latter influences virtually all biological processes).

At non-extreme temperatures, the folded over total proteins ratio *η*(*T*) is almost constant and close to one (see Supplementary Fig. 4A), i.e., unfolding is negligible with respect to folding. So temperature has only an effect on the metabolic and translational rates. We then obtain a linear relationship between the Arrhenius-corrected growth rate and the ribosome content:

10 $${r}_{tot}\simeq \frac{1}{{k}_{R}^{ref}}\nu +{r}_{0}.$$

Thus, in this temperature range, the Arrhenius-corrected growth rate follows directly from the active ribosome content, given that almost all the proteins are folded, and that optimal allocation leads to constant ribosome efficiency.

**Fig. 4:**
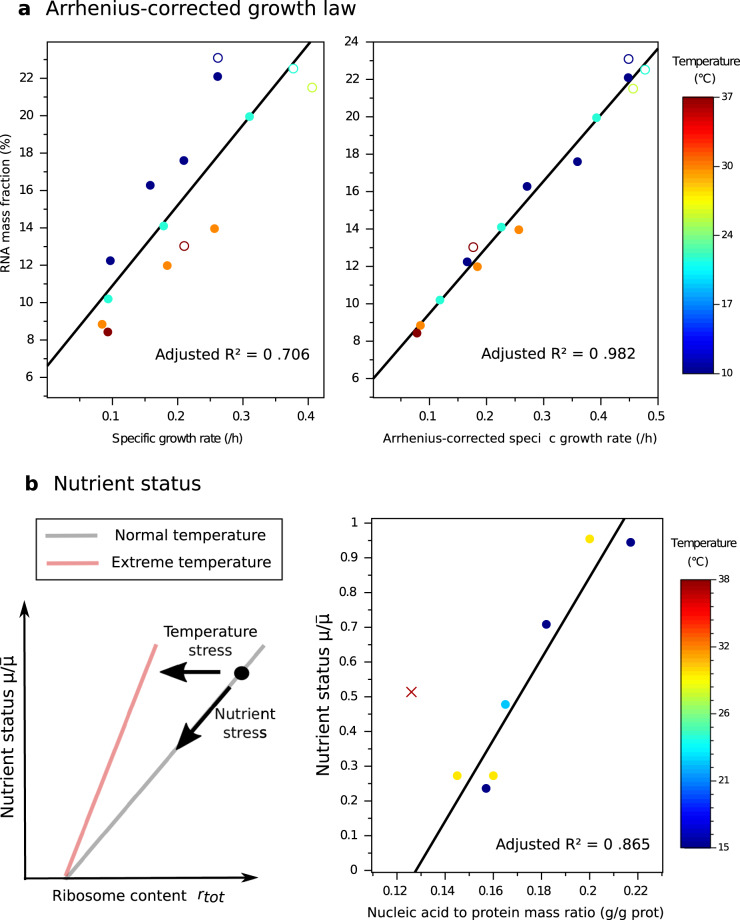
Microbial growth laws accounting for temperature effects. **a** Growth law between growth rate and RNA content, without (left) and with (right) Arrhenius correction (Eqs. (9)–(10)) for *S. cerevisiae*^24^. The Arrhenius correction greatly improves the linear regression, as witnessed by the increase of adjusted R^2^ from 0.706 to 0.982. Open symbols: batch; closed symbols: chemostat. For each point, color represents temperature (see colorbar). **b** Nutrient status as a function of the ribosome content. Left: the gray line represents the theoretical relationship valid for non-extreme temperatures (from Eq. (12)). At extreme temperatures, the slope changes due to chaperone burden (Eq. (11)). A decrease in ribosome content relative to a control (black dot) could be due to a substrate limitation and/or temperature stress. Right: nutrient status as a function of the nucleic acid to protein ratios for *Candida utilis*^43^. The only point above the optimal temperature for growth, marked by x, was identified as an outlier and removed from the linear regression, showing a possible effect of temperature stress in line with our prediction.

The Arrhenius-corrected growth law is confirmed in Fig. 4A by means of experimental data for *S. cerevisiae*^24^, obtained at different temperatures and levels of substrate limitation (using RNA as a proxy for *r*_*tot*_). While the temperature clearly disrupts the linear relationships between ribosome content and specific growth rate, the Arrhenius correction allows a robust linear relationship to be recovered over a wide range of temperatures.

Using the Arrhenius-corrected growth law (10), the growth rate of a species can be theoretically estimated from the measurements of temperature and its ribosome content:

$$\mu ={k}_{R}^{ref}\exp \left(\frac{-{E}_{R}}{R}\left(\frac{1}{T}-\frac{1}{{T}_{ref}}\right)\right)({r}_{tot}-{r}_{0}),$$

without completely recalibrating the model of Eqs (1)–(4). More precisely, one must know the maximum protein synthesis rate ${k}_{R}^{ref}$, the inactive ribosomal mass fraction *r*_0_ and the activation energy *E*_*R*_. The latter has been estimated from thermal growth curves for a large set of species^42^, and a mean value of 0.63 eV has been proposed for temperature correction of metabolic rates^41^. The rate ${k}_{R}^{ref}$ can be estimated from the maximum protein elongation rate^5^, while the minimum ribosome content *r*_0_ has also been measured for model species^28,29^. Experimental validation of the Arrhenius-corrected growth law on more species would be valuable to assess the variability of the aforementioned parameters.

Based on optimality principles, we have thus extended the growth law relating ribosome content to growth rate^9^ to the case of different temperatures. Translation rate and protein stability are affected by temperature. Under non-stressful conditions, we could consider only the Arrhenius effect, leading to a linear relationship between the ribosome content and the Arrhenius-corrected growth rate of Eq. (10). Under stressful conditions, a non-negligible part of the proteins are denatured, and the chaperone burden diverts resources away from growth. This results in the more complex growth law given by Eq. (7).

### Determining the nutrient status from the optimal resource allocation

Measurements of ribosome content (or equivalently the RNA/DNA ratio) are used in ecology to estimate growth rates or cellular activities of microorganisms. We have seen theoretically how to correct this estimation for temperature. However, not all species are well characterized, and field measurements are generally more qualitative than quantitative, which may make the estimation of growth rate questionable. Exploiting the optimal resource allocation in a different way, we could instead determine the nutrient status from the ribosome content. This could provide a more reliable and relevant indicator of microbial ecosystem functioning than growth rate or activity.

Let $\bar{\mu }(T)$ be the maximal growth rate at a given temperature. We call the ratio $\mu /\bar{\mu }$ the “nutrient status”, as it reflects how substrate availability limits growth in comparison with non-limiting conditions. Whenever the activation energy is the same for the metabolic and translational reactions, we can formulate the following relationship between the nutrient status $\mu /\bar{\mu }$ and the mass fraction of ribosomes *r*_*tot*_ and chaperones *c* (see Method for details):

11 $$\frac{\mu }{\bar{\mu }}=\frac{1-q}{1-q-c}\,\frac{{r}_{tot}-{r}_{0}}{{r}_{max}-{r}_{0}}$$

with *r*_*max*_ a constant (given in Method), corresponding to the hypothetical ribosome content for a saturating substrate concentration in the case of perfect protein folding (without chaperones). From a practical point of view, *r*_*max*_ approximately corresponds to the maximal ribosome content of the species obtained at a non-stressful temperature (given that chaperones are always present, but in negligible quantity).

The relationship expressed by Eq. (11) is more difficult to validate by experimental data, given that it requires a precise quantification of *r*_*tot*_ and *c*. However, following the same simplification as for the growth law, at non-extreme temperatures, the chaperone mass fraction *c* is almost constant and very small compared to 1 − *q*. We thus end up with an approximate linear relationship between nutrient status $\mu /\bar{\mu }$ and ribosome content (see Fig. 4B, left):

12 $$\frac{\mu }{\bar{\mu }}\simeq \frac{{r}_{tot}-{r}_{0}}{{r}_{max}-{r}_{0}}.$$

This equation shows that, in absence of temperature stress, the comparison of the ribosome (or RNA) content allows a direct characterization of the nutrient status of a species. The use of Eq. (12) does not require any model calibration nor knowledge of the species, which makes it much easier to use than the temperature-corrected growth law given by Eq. (10). For a quantitative estimation of the nutrient status with Eq. (12), only the parameters *r*_0_ and *r*_*max*_—corresponding to the minimum and maximum ribosome contents—have to be estimated from experimental data.

Such a linear relationship has been observed experimentally for the yeast *Candida utilis*^43^. In chemostat steady states at different dilution rates and temperatures, the authors observed a linear dependence between the nucleic acid to protein ratios and the nutrient status, in line with our theoretical prediction (see Fig. 4B, right). By re-analyzing their data, however, we identified one point as an outlier (with a Cook’s distance of 0.98^44^) and excluded it from the linear regression. This point (at 37.5 ^∘^C) is the only measurement taken above the optimal temperature for growth. The theoretical development above suggests that the deviation is not a measurement anomaly, but due to protein denaturation and chaperone burden (Fig. 4B, left). Once again, this example shows that stress, if it exists, must be taken into account in the prediction of resource allocation and the analysis of environmental data.

In conclusion, our coarse-grained approach has allowed us to derive an environmental indicator, based on the linear relationship between ribosome content and nutrient status for non-stressful temperatures (Eq. (12)), which is convenient to use in practice. It is important to remind though that the slope of the linear relationship changes at extreme temperatures, due to the chaperone burden.

## Discussion

Based on a coarse-grained model, we have investigated how optimal resource allocation of microorganisms varies with substrate availability and temperature. The optimal allocation of resources to different protein sectors agrees well with quantitative proteomic data. The model has allowed us to account for temperature-induced changes in physiology (growth) in terms of variations in ribosomal and chaperone contents, consistent with experimental data.

At non-extreme temperatures, protein unfolding is negligible, and the metabolic and translational rates were found to follow the same temperature dependence given by the Arrhenius law (*E*_*M*_ = *E*_*R*_). As a consequence, optimal resource allocation is (almost) constant, while the specific growth rate increases with temperature. That is, readjustment within the cell is not necessary given that the increase in temperature affects all reactions uniformly. In addition, the optimal allocation leads to precursor saturation of active ribosomes, which finally gives a linear relationship between the ribosome content and the Arrhenius-corrected growth rate (Eq. (10)). This generalizes the so-called microbial growth law^9^ to different temperatures. A variant of this extended growth law establishes a linear relationship between the ribosome content and the nutrient status (Eq. (12)).

On the contrary, in the case of extreme (high or low) temperatures, the resource allocation profile changes to cope with protein denaturation, through the synthesis of chaperones. This leads to a trade-off between chaperone cost versus benefit in order to maximize the pool of folded proteins, and ultimately the growth rate. This chaperone burden leads to a decrease of ribosomal and metabolic proteins at extreme temperatures, as captured by the more complex, Arrhenius-corrected growth law of Eq. (7).

Several mechanistic models have been proposed to decipher the effects of temperature or stress on microbial growth through protein folding and unfolding^17,21,30,45,46^. Here, we choose a minimal model, which allows to obtain analytical results and to derive growth laws. Our approach is based on a set of reasonable assumptions and classic elements from coarse-grained models of bacterial growth^3,5^ and protein folding^17^.

In biology, many predictions from first principles rely on optimization approaches^47,48^. A cornerstone of optimization approaches is the choice of an appropriate objective criterion. In our development, the main hypothesis is that microbial resource allocation has evolved such that it maximizes growth rate at balanced growth for all temperature and nutrient conditions. This agrees with the observation that strains are adapted to a range of temperatures, generally below their optimal temperature for growth^49^. However, in adaptive laboratory evolution experiments at different temperatures, fitness increases have been observed^50^, involving regulatory processes that potentially affect resource allocation^51–53^. Although resource allocation may therefore not be optimal over the whole temperature range, the remarkable agreement of predictions and experimental data presented here nevertheless shows that the underlying hypotheses are a valid starting-point.

With respect to the effect of temperature, we assume that all protein sectors have the same folding-unfolding kinetics, and the best model (in terms of AIC) was obtained when assuming that the metabolic and translational reactions respond with the same increase. The constant cellular composition of *E. coli* and *S. cerevisiae* over a range of non-extreme temperatures, correctly predicted by the model, is consistent with this model assumption. Note that equal activation energies for metabolic enzymes and ribosomes at the coarse-grained level does not imply that individual proteins in these sectors need to have the same temperature sensitivities. Within each sector, adjustments in the amount of some proteins probably take place to compensate for different temperature sensitivities, without affecting the overall sector allocation. In a more detailed analysis, it would be possible to assign different thermal sensitivities to some pathways or reactions. Such an extension could be based on the skillful experiments by Chang et al.^12^ in which nutrient supplementation was used to bypass temperature-sensitive enzymes. Another striking case concerns photosynthetic microorganisms, for which we know that the light phase of photosynthesis is less affected by temperature than other cellular processes^54^. As a consequence, resource allocation varies, even at the coarse-grained level, over the entire temperature range^55^. In order to account for temperature dependence in these microorganisms, using the same activation energy for the metabolic and translational rates is not possible, and thus the derivation of the growth laws would need to be revisited.

In our framework, misfolded proteins represent wasted resources, but the reality is even worse in that misfolded proteins cause cellular damage^13^. Including the deleterious effects of misfolded proteins in the model will probably increase the optimal chaperone content at extreme temperatures to keep unfolded proteins at a lower level. The degradation of misfolded proteins is also an important cellular process^56^ and could be included in the model^57^, leading to further small adjustments of optimal proteome allocation.

A precise prediction of resource allocation in any temperature or substrate condition is a real challenge. Despite the above-mentioned limitations, our coarse-grained approach is capable of predicting and explaining a large variety of experimentally observed resource allocation patterns. Moreover, we show how the temperature dependence of resource allocation can be easily integrated in generalized growth laws. This is of utmost importance when, for example, ribosomal RNA is used in ecology to evaluate microbial growth, and it could explain some observed conflicting patterns^23^ given that temperature correction is generally omitted.

As an example of this point, the rRNA/rDNA ratio of different heterotrophic picoeukaryotes present all over the water column have been computed by Giner et al.^58^ for each identified Operational Taxonomic Units from the high-throughput sequencing of the 18S rRNA gene from DNA and RNA extracts. They have observed that this ratio is generally higher in the mesopelagic layer (200–1000 m depth) than in the epipelagic (0–200 m) and bathypelagic (1000–4000 m) layers. This has led them to conclude that the activity of these microorganisms follows the same pattern. Given that temperature decreases with depth, however, our analysis suggests that this conclusion may be premature. Actually, a low ribosome content at high temperature can give a higher activity than a high ribosome content at low temperature. Based on the principle of nutrient status illustrated in Fig. 4B, we could infer that the low RNA content at the surface reflects the effect of substrate limitation in comparison with the mesopelagic layer, whereas the low RNA content in the deep ocean reflects temperature stress. This is in line with the presence of organic matter in the the mesopelagic layer, and the temperature of around 3 ^∘^C in the bathypelagic layer^58^.

More generally, to characterize ecosystem functioning over a range of temperatures in the absence of heat or cold stress, we propose to estimate from the ribosome (or RNA) content the nutrient status (Eq. (12)), rather than the microbial activity or growth rate. Given that this nutrient status is not affected by temperature over a fairly wide range and requires less information for its application than the Arrhenius-corrected growth law of Eq. (10), this indicator is more robust for environmental studies. Even in the absence of quantitative information on the minimum and maximum ribosome contents, it allows a qualitative comparison of relative nutrient status of a species over several conditions with non-extreme temperatures. Our results thus illustrate the benefit of coarse-grained models and optimization approaches for unraveling complex ecological systems.

## Methods

### Computation of optimal allocation

Resource allocation is computed as the solution of the optimization problem under constraints (5). The constraints

$$\frac{dc}{dt}=\frac{dm}{dt}=\frac{dr}{dt}=0$$

directly give

$${\alpha }_{c}^{opt}\ =\ {c}^{opt},\ {\alpha }_{r}^{opt}\ =\ {r}^{opt},\ {\rm{and}}\ \ {\alpha }_{m}^{opt}\ =\ {m}^{opt}.$$

Thus, (5) can be rewritten as:

$$\begin{array}{ll}({c}^{opt},{r}^{opt},{p}^{opt})=&\mathop{{\mathrm{arg}}\,{\mathrm{max}}}\limits_{c,r,p}\ \mu \\ &{\rm{such}}\ {\rm{that}}\ \frac{dp}{dt}=0,\end{array}$$

with *m* = 1 − *q* − *r* − *c*. The problem is solved using the method of Lagrange multipliers^59^. Let us call $F=\mu -\lambda \frac{dp}{dt}$, with *λ* the Lagrange multiplier. The solution of (5) is determined by solving the following system:

13 $$\left\{\begin{array}{l}\frac{\partial F}{\partial c}\,=\,{k}_{R}{k}_{f}{k}_{u}\frac{p}{{K}_{p}+p}\frac{r}{{({k}_{u}+{k}_{f}c)}^{2}}+\lambda {k}_{M}g(s)=0\\ \frac{\partial F}{\partial r}\,=\,{k}_{R}\frac{p}{{K}_{p}+p}\frac{{k}_{f}c}{{k}_{u}+{k}_{f}c}+\lambda \left[{k}_{M}g(s)+{k}_{R}(1+p)\frac{p}{{K}_{p}+p}\right]=0\\ \frac{\partial F}{\partial p}\,=\,{k}_{R}\frac{{K}_{p}}{{({K}_{p}+p)}^{2}}\frac{{k}_{f}c}{{k}_{u}+{k}_{f}c}r+\lambda {k}_{R}r\frac{{K}_{p}+2{K}_{p}p+{p}^{2}}{{({K}_{p}+p)}^{2}}=0\\ \frac{\partial F}{\partial \lambda }\,=\,{k}_{M}g(s)(1-r-c-q)-(1+p){k}_{R}\frac{p}{{K}_{p}+p}r=0\\ \end{array}\right.$$

which gives (6) after some algebraic manipulations, including solving a second degree equation.

### Model calibration

Model parameters are estimated with three experimental data sets concerning *E. coli*:

- Two thermal response curves, i.e., specific growth rates obtained in non-limiting conditions at different temperatures, in glucose minimal medium^27^ and in rich medium^26^.
- Quantitative proteomic data for seven conditions^25^: four chemostat steady states at 37 ^∘^C with different dilution rates (0.12, 0.2, 0.35, and 0.5/h), and three cultures in batch (sampled during the exponential growth phase) at 37 and 42 ^∘^C in glucose minimal medium, and at 37 ^∘^C in rich medium. The following proteins have been considered to compute the proteome sectors:
  – for the metabolic proteins *m*: the Clusters of Orthologous Groups (COGs) “Amino acid transport and metabolism”, “Energy production and conversion”, and “Carbohydrate transport and metabolism”, which include all reactions going from substrate uptake to the production of amino acids.
  – for the ribosomal proteins *r*: the COG “Translation, ribosomal structure and biogenesis”.
  – for the chaperones *c*: a subset of the COG “Post-translational modification, protein turnover, chaperones”, notably the proteins encoded by the genes *groL, dnaK, htpG, ppiD, surA, grpE, hscA, dnaJ, fkpA, hslO, ppiA, hscB, cbpA, ppiB, slyD, ybbN, groS, fklB, clpB, ibpA, ppiC, ibpB, fkpB, yegD, dsbB, djlA, clpB, ynfD, yoaC, yqjK, yrbL, cgtA, gatR, tusA, wecA*.

Note that *groL* and *dnaK* corresponds to two-thirds of the total chaperone mass on average.

Two parameters have not been included in the calibration due to identifiability issues:

- The half-saturation constant for protein synthesis *K*_*p*_ has a low sensitivity on model fitting, given that it affects principally *p*^*opt*^ which has not been measured. The value proposed in ref. ^5^ has therefore been used.
- Given that model outputs depend only on the ratio ${k}_{f}^{ref}/{k}_{u}^{ref}$, we identify this ratio rather than the two parameters individually.

In addition, the inactive ribosomal content *r*_0_ has been computed directly from the experimental data set of ref. ^25^. The house-keeping protein content *q* is taken as the mean of 1 − *m* − *r* − *c* from the same dataset, recalling that *r* = *r*_*t*_ − *r*_0_, i.e., *r*_0_ is included in *q*. Finally, two values for the maximum synthesis rate for precursors have been identified for glucose-minimal medium and for rich medium, called ${k}_{M}^{ref}$ and ${\tilde{k}}_{M}^{ref}$, respectively.

Parameter values have been estimated by minimizing the sum of squared errors between model outputs and measurements, with the Levenberg-Marquardt algorithm (implemented in the lmfit toolbox^60^ in Python). The algorithm has been initialized with parameter reference values determined from literature. For chemostat data, given that the substrate concentration is unknown, the optimal allocation (and the corresponding growth rate) are computed for *g*(*s*) ranging from 0 to 1. The solutions are then interpolated to compute the allocations with growth rates equal to the dilution rates. After a first fit with three different activation energies for precursor and protein synthesis and protein folding, further parameter estimation runs with additional constraints on these activation energies (to reduce the number of parameter) have been carried out (see Table 1). The best fit in terms of AIC is obtained when the metabolic reactions have the same activation energy (*E*_*M*_ = *E*_*R*_), which removes one parameter while the residual sum of square hardly increases.

**Table 1:** Fit statistics for the coarse-grained resource allocation model.

Constraint	−	*E*_*M*_ = *E*_*R*_	*E*_*R*_ = *E*_*f*_	*E*_*M*_ = *E*_*f*_	*E*_*M*_ = *E*_*R*_ = *E*_*f*_
Number of parameters	9	8	8	8	7
Residual sum of square	1.74	1.76	2.18	2.44	2.48
AIC	−150.0	−151.3	−140.7	−134.9	−136.2

To find the best trade-off between model simplicity and accuracy, we evaluate if different activation energies should be considered for the Arrhenius functions.

We have further explored the parameter space for the model with *E*_*M*_ = *E*_*R*_ to determine the probability distributions for the parameters and their correlations using a Monte-Carlo Markov Chain algorithm. More precisely, the affine invariant sampling algorithm emcee^61^ (from the lmfit toolbox^60^) has been used with 200 walkers, initialized with the parameter values obtained with the Levenberg-Marquardt algorithm.

Identified values (given in Table 2) are globally close to the reference values, with relatively low standard error. The two parameters describing the effect of temperature on protein folding (in *φ*_*f*_(*T*)) and unfolding (in *ψ*(*T*)), namely *E*_*f*_ and *E*_*u*_, present the larger variations. Actually, these two parameters are clearly correlated, as revealed by their posterior distributions shown in Supplementary Fig. 6. Despite parameter variations, the functions *φ*_*f*_(*T*) and *ψ*(*T*) with the initial guess or with the identified parameters are relatively close in the temperature range of interest (see Supplementary Fig 4B).

**Table 2:** Parameter values for the coarse-grained resource allocation model.

Parameter	Symbol	Reference value (source)	Calibrated value	Units
Maximum synthesis rate for precursors				
in glucose-minimum medium	${k}_{M}^{ref}$	3.6 (a)	1.54 ± 0.12	/h
in rich medium	${\tilde{k}}_{M}^{ref}$	3.6 (a)	9.99 ± 1.61	/h
Maximum synthesis rate for proteins	${k}_{R}^{ref}$	3.6 (a)	8.42 ± 1.13	/h
Protein folding/unfolding rate ratio	${k}_{f}^{ref}/{k}_{u}^{ref}$	200 (b)	357 ± 60	h/h
Activation energy for the synthesis rates	*E*_*M*_, *E*_*R*_	45,000 (c)	41,900 ± 5,200	J/mol
Activation energy for protein folding	*E*_*f*_	84,000 (d)	489,000 ± 81,000	J/mol
Pseudo activation energy for protein unfolding	*E*_*u*_	36,400 (e)	575,000 ± 97,000	J/mol
Deformation parameter for protein unfolding	*d*	−0.033 (e)	−0.094 ± 0.025	–
Half-saturation constant for protein synthesis	*K*_*p*_	0.003 (a)	–	g/g
House-keeping protein mass fraction	*q*	0.46 (f)	–	g/g
Inactive ribosomal protein mass fraction	*r*_0_	0.138 (f)	–	g/g

a: from ref. ^5^; b: from ref. ^17^; c: computed from ref. ^37^; d: from ref. ^33^; e: computed from ref. ^62^; f: computed from ref. ^25^.

Parameters were fitted to experimental data shown in Fig. 2. For the last three parameters, values were taken from literature. Kinetic rates are given at Tref = 310.15 K.

### Determination of growth laws

We use the solution of the optimal resource allocation problem, given by (6), to derive the link between growth rate and ribosome content. For sake of brevity, we omit the superscript *opt* in the following.

The optimal growth rate, as an explicit function of temperature and substrate concentration, is given by:

$$\mu (T,s)={k}_{R}(T)\frac{p(s)}{{K}_{p}+p(s)}{r}_{f}(T,s).$$

We call *η* the fraction of folded proteins:

$$\eta =\frac{{r}_{f}}{r}=\frac{{m}_{f}}{m}=\frac{{q}_{f}}{q}.$$

From Eq. (2), we directly get

$$\eta =\frac{{k}_{f}(T)c}{{k}_{u}(T)+{k}_{f}(T)c}.$$

Using the expression for *c* (Eq. (6)), *η* depends only on temperature:

$$\eta (T)=1-{\left[1+(1-q)\frac{{k}_{f}(T)}{{k}_{u}(T)}\right]}^{-1/2},$$

and so we have

14 $$\mu (T,s)={k}_{R}(T)\frac{p(s)}{{K}_{p}+p(s)}\eta (T)r(T,s).$$

If the medium is not particularly scarce, i.e., if *g*(*s*) = *s*/(*K*_*s*_ + *s*) ≫ *K*_*p*_*k*_*R*_/*k*_*M*_, then *p*(*s*) ≫ *K*_*p*_ so the second term in the right-hand side of the previous equation is almost equal to one. This corresponds to precursor saturation, leading to a constant translation rate (or ribosome efficiency) of active ribosomes, in line with experimental observations^39,40^. Recalling that the total ribosomal sector is the sum of the optimal ribosome content plus the inactive reserve^28^:

$${r}_{tot}=r+{r}_{0},$$

we obtain a linear relationship between *μ* and *r*_*tot*_ for a constant temperature. Nonetheless, the slope depends on temperature:

$${r}_{tot}=\frac{1}{{k}_{R}(T)\eta (T)}\mu +{r}_{0}.$$

### Determination of the nutrient status

The solution of the optimal resource allocation problem can also be used to investigate the relationship between the nutrient status and the ribosome content.

Call $\bar{\mu }(T)$ the maximal growth rate at a given temperature, obtained for a saturating substrate concentration, i.e., $g(s)=\frac{s}{{K}_{s}+s}\approx 1$, and $\bar{p}$ and $\bar{r}{(T)}$ the corresponding mass fractions of precursors and ribosomes, respectively. From Eq. (6) with *E*_*M*_ = *E*_*R*_, $\bar{p}$ is independent of temperature and given by

$$\bar{p}=\sqrt{\frac{{k}_{M}^{ref}{K}_{p}}{{k}_{R}^{ref}}}.$$

Using Eq. (14), the nutrient status $\mu /\bar{\mu }$ is given by

$$\begin{array}{lll}\frac{\mu (T,s)}{\bar{\mu (T)}}&=&\frac{{k}_{R}(T)\frac{p(s)}{{K}_{p}+p(s)}\eta (T)r(T,s)}{{k}_{R}(T)\frac{\bar{p}}{{K}_{p}+\bar{p}}\eta (T)\bar{r}{(T)}}\\ &=&\frac{p(s)}{{K}_{p}+p(s)}\frac{{K}_{p}+\bar{p}}{\bar{p}}\frac{r(T,s)}{\bar{r}{(T)}}.\end{array}$$

In the latter equation, the two first terms in the right-hand side are almost one (assuming precursor saturation, as stated before), so the nutrient status is almost equal to the ribosome content ratio. From Eq. (6), the active ribosome mass fraction for a saturating substrate concentration $\bar{r}{(T)}$ is

$$\bar{r}{(T)}=(1-q-c(T))\frac{{\bar{p}}(K_{P}+\bar{p})}{{K}_{p}+2{K}_{p}\bar{p}+{\bar{p}}^{2}}.$$

Note that the dependence on temperature only comes from *c*(*T*). Recalling that *r* = *r*_*tot*_ − *r*_0_, let *r*_*max*_ be the hypothetical ribosome content for a saturating substrate concentration with perfect protein folding (without the need of chaperones):

$${r}_{max}={r}_{0}\,+\,(1-q)\frac{\bar{p}({{K}_{p}}+\bar{p})}{{K}_{p}+2{K}_{p}\bar{p}+{\bar{p}}^{2}},$$

so that ${\bar{r}}{(T)}$ becomes

$$\bar{r}{(T)}=\frac{1-q-c(T)}{1-q}({r}_{max}-{r}_{0}).$$

We finally get the following relationship between the nutrient status and the ribosome mass fraction:

$$\frac{\mu }{\bar{\mu }}=\frac{1-q}{1-q-c(T)}\ \frac{{r}_{tot}-{r}_{0}}{{r}_{max}-{r}_{0}}.$$

### Reporting summary

Further information on research design is available in the Nature Research Reporting Summary linked to this article.

## Supplementary information

Supplementary material

reporting summary

## Data Availability

This study did not generate datasets.

## Code availability

The code for parameter estimation is available upon request. The code for model simulation (Figs. 2 and 3) is available at: https://github.com/fmairet/Temperature_Allocation.
