# Supplementary material for: Optimal proteome allocation and the temperature dependence of microbial growth laws
Source: NPJ Syst Biol Appl. 2021 Mar 8;7:14. doi: 10.1038/s41540-021-00172-y (PMC7940435; doi:10.1038/s41540-021-00172-y)
Supplement: Supplementary file 1 — Supplementary material [file 41540_2021_172_MOESM1_ESM.pdf]

Supplementary Material for "Optimal proteome allocation and the temperature dependence of microbial growth laws"

Francis Mairet, Jean-Luc Gouzé, Hidde de Jong

1 Model development

Let P, C, M, Q, and R be the total mass (in g) of precursors, chaperones, metabolic proteins, house-keeping proteins, and ribosomal proteins, respectively. Metabolic, ribosomal, and house-keeping proteins are either folded or unfolded (denoted respectively with subscript f and u):

$$M = M_f + M_u, \quad R = R_f + R_u, \quad \text{and} \quad Q = Q_f + Q_u.$$

Biomass B (in g prot) is defined as the total mass of proteins:

$$B = C + M + Q + R.$$

Biochemical reactions

We consider the following reactions (represented in Figure 1 of the main article):

- Precursor synthesis:
$$\mathrm{S} \xrightarrow{v_M} \mathrm{P},$$
where S is the substrate, and v_M is the rate of precursor synthesis per unit of biomass (in g/(g prot·h)).

- Protein synthesis:
$$\mathrm{P} \xrightarrow{v_R} \alpha_c \mathrm{C} + \alpha_m \mathrm{M}_u + \alpha_q \mathrm{Q}_u + \alpha_r \mathrm{R}_u,$$
where v_R is the rate of protein synthesis per unit of biomass (in g/(g prot·h)), and $\alpha_c, \alpha_m, \alpha_q$, and α_r are the allocation variables, between 0 and 1 with their sum equal to 1.

- Protein folding:
$$\mathrm{M}_u \xrightarrow{v_f^m} \mathrm{M}_f,$$
$$\mathrm{Q}_u \xrightarrow{v_f^q} \mathrm{Q}_f,$$
$$\mathrm{R}_u \xrightarrow{v_f^r} \mathrm{R}_f,$$

where v_f^m, v_f^r, and v_f^q are the folding rates per unit of biomass (in g/(g prot·h)) of metabolic proteins, house-keeping proteins, and ribosomal proteins, respectively.

- Protein unfolding:

$$\mathrm{M}_f \xrightarrow{v_u^m} \mathrm{M}_u,$$
$$\mathrm{Q}_f \xrightarrow{v_u^q} \mathrm{Q}_u,$$
$$\mathrm{R}_f \xrightarrow{v_u^r} \mathrm{R}_u,$$

where v_u^m, v_u^r, and v_u^q are the unfolding rates per unit of biomass (in g/(g prot·h)) of metabolic proteins, house-keeping proteins, and ribosomal proteins, respectively.

Mass balance

Given this set of reactions, we obtain the following mass-balanced system:

$$\begin{cases} \dfrac{dP}{dt} &= v_M B - v_R B, \\ \dfrac{dC}{dt} &= \alpha_c v_R B, \\ \dfrac{dM}{dt} &= \alpha_m v_R B, \\ \dfrac{dR}{dt} &= \alpha_r v_R B, \\ \dfrac{dQ}{dt} &= \alpha_q v_R B, \\ \dfrac{dM_f}{dt} &= v_f^m B - v_u^m B, \\ \dfrac{dR_f}{dt} &= v_f^r B - v_u^r B, \\ \dfrac{dQ_f}{dt} &= v_f^q B - v_u^q B. \end{cases} \tag{1}$$

Kinetics

Assuming that the mass of precursors is negligible compared to that of proteins, we define the mass fractions (in g/g prot) as follows:

$$p = P/B, \quad c = C/B, \quad m = M/B, \quad q = Q/B, \quad r = R/B.$$

The rates for precursor and protein synthesis are taken as Michaelis-Menten functions:

$$v_M(s, m_f) = k_M \frac{s}{K_s + s} m_f, \qquad \text{and} \qquad v_R(p, r_f) = k_R \frac{p}{K_p + p} r_f.$$

Folding and unfolding rates are represented by mass-action kinetics:

$$v_f^m(m_u, c) = k_f m_u c, \qquad v_f^r(r_u, c) = k_f r_u c, \qquad v_f^q(q_u, c) = k_f q_u c,$$

and

$$v_u^m(m_f) = k_u m_f, \qquad v_u^r(r_f) = k_u r_f, \qquad v_u^q(q_f) = k_u q_f.$$

Finally, we can compute the specific growth rate μ:

$$\mu = \frac{1}{B}\frac{dB}{dt} = \frac{1}{B}(\alpha_c + \alpha_m + \alpha_r + \alpha_q)v_R B = v_R.$$

Actually, given that biomass corresponds by definition to proteins, the specific growth rate equals the rate of protein synthesis per unit of biomass.

Full kinetic model

We get by simple derivation the dynamics of the mass fractions:

$$\begin{cases} \frac{dp}{dt} &= \frac{1}{B}\frac{dP}{dt} - \frac{P}{B^2}\frac{dB}{dt} &= v_M(s, m_f) - (1+p)v_R(p, r_f), \\ \frac{dc}{dt} &= \frac{1}{B}\frac{dC}{dt} - \frac{C}{B^2}\frac{dB}{dt} &= (\alpha_c - c)v_R(p, r_f), \\ \frac{dm}{dt} &= \frac{1}{B}\frac{dM}{dt} - \frac{M}{B^2}\frac{dB}{dt} &= (\alpha_m - m)v_R(p, r_f), \\ \frac{dr}{dt} &= \frac{1}{B}\frac{dR}{dt} - \frac{R}{B^2}\frac{dB}{dt} &= (\alpha_r - r)v_R(p, r_f), \\ \frac{dq}{dt} &= \frac{1}{B}\frac{dQ}{dt} - \frac{Q}{B^2}\frac{dB}{dt} &= (\alpha_q - q)v_R(p, r_f), \\ \frac{dm_f}{dt} &= \frac{1}{B}\frac{dM_f}{dt} - \frac{M_f}{B^2}\frac{dB}{dt} &= v_f^m(m_u, c) - v_u^m(m_f) - m_f v_R(p, r_f), \\ \frac{dr_f}{dt} &= \frac{1}{B}\frac{dR_f}{dt} - \frac{R_f}{B^2}\frac{dB}{dt} &= v_f^r(r_u, c) - v_u^r(r_f) - r_f v_R(p, r_f), \\ \frac{dq_f}{dt} &= \frac{1}{B}\frac{dQ_f}{dt} - \frac{Q_f}{B^2}\frac{dB}{dt} &= v_f^q(q_u, c) - v_u^q(q_f) - q_f v_R(p, r_f). \end{cases} \tag{2}$$

Quasi-steady-state approximation (QSSA)

The folding/unfolding rates are much faster than the synthesis rates of precursors and proteins [1]. Therefore, the system can be decomposed into two time-scales: the first five equations are slow, while the last three ones are fast. Using the quasi-steady-state assumption [2], the system can be reduced to the dynamics of the slow system, taking the fast system at equilibrium. Neglecting the small terms ($v_r << v_f, v_u$), the equilibrium of the fast system is given by

$$\begin{cases} v_f^m = v_u^m, \\ v_f^r = v_u^r, \\ v_f^q = v_u^q. \end{cases} \tag{3}$$

Using the aforementioned kinetic expressions, the fast equilibrium $v_f^m = v_u^m$ gives:

$$k_f m_u c = k_u m_f.$$

Recalling that $m = m_u + m_f$, we finally get:

$$m_f = \frac{k_f c}{k_u + k_f c} m.$$

Similarly, we obtain for the other protein sectors:

$$r_f = \frac{k_f c}{k_u + k_f c} r \quad \text{and} \quad q_f = \frac{k_f c}{k_u + k_f c} q.$$

With this approximation, we obtain the QSSA model presented in the main text. Plugging in the kinetic expressions, the system reads:

$$\begin{cases} \dfrac{dp}{dt} & = \dfrac{k_f(T)c}{k_u(T) + k_f(T)c} \left[k_M(T) \dfrac{s}{K_s + s} m - (1+p) k_R(T) \dfrac{p}{K_p + p} r \right], \\ \dfrac{dc}{dt} & = (\alpha_c - c) k_R(T) \dfrac{p}{K_p + p} \dfrac{k_f(T)c}{k_u(T) + k_f(T)c} r, \\ \dfrac{dm}{dt} & = (\alpha_m - m) k_R(T) \dfrac{p}{K_p + p} \dfrac{k_f(T)c}{k_u(T) + k_f(T)c} r, \\ \dfrac{dr}{dt} & = (\alpha_r - r) k_R(T) \dfrac{p}{K_p + p} \dfrac{k_f(T)c}{k_u(T) + k_f(T)c} r, \\ \dfrac{dq}{dt} & = (\alpha_q - q) k_R(T) \dfrac{p}{K_p + p} \dfrac{k_f(T)c}{k_u(T) + k_f(T)c} r. \end{cases} \tag{4}$$

Comparison of the full model and the QSSA model

Finally, we compare the optimal solutions of System (4) (obtained analytically) and of System (2) (obtained numerically), using the parameter set given in Tab. 2 of the main article. Given that only the ratio k_f/k_u has been estimated, the value of k_f has to be fixed. We consider two cases:

- k_f is such that $\frac{v_f}{v_R} \simeq 100$. This corresponds to the difference between the mean rates of protein folding and synthesis [1], which supports our slow-fast approximation.
- k_f is such that $\frac{v_f}{v_R} \simeq 1$, corresponding to a worst case with no time-scale separation.

In the first case, the equilibria of the QSSA system are almost identical to those of the complete system (Supplementary Fig. 5B). In the second case, the equilibria are different, but the trends remain similar. The QSSA allows to obtain a simpler model (much more tractable for mathematical analysis) with almost the same optimal equilibria, which supports our approach. Nonetheless, given the wide time scale distribution of protein folding rates [1], the QSSA model should be used with caution in other situations, *e.g.*, to study short-term response and regulation.

2 Supplementary figures

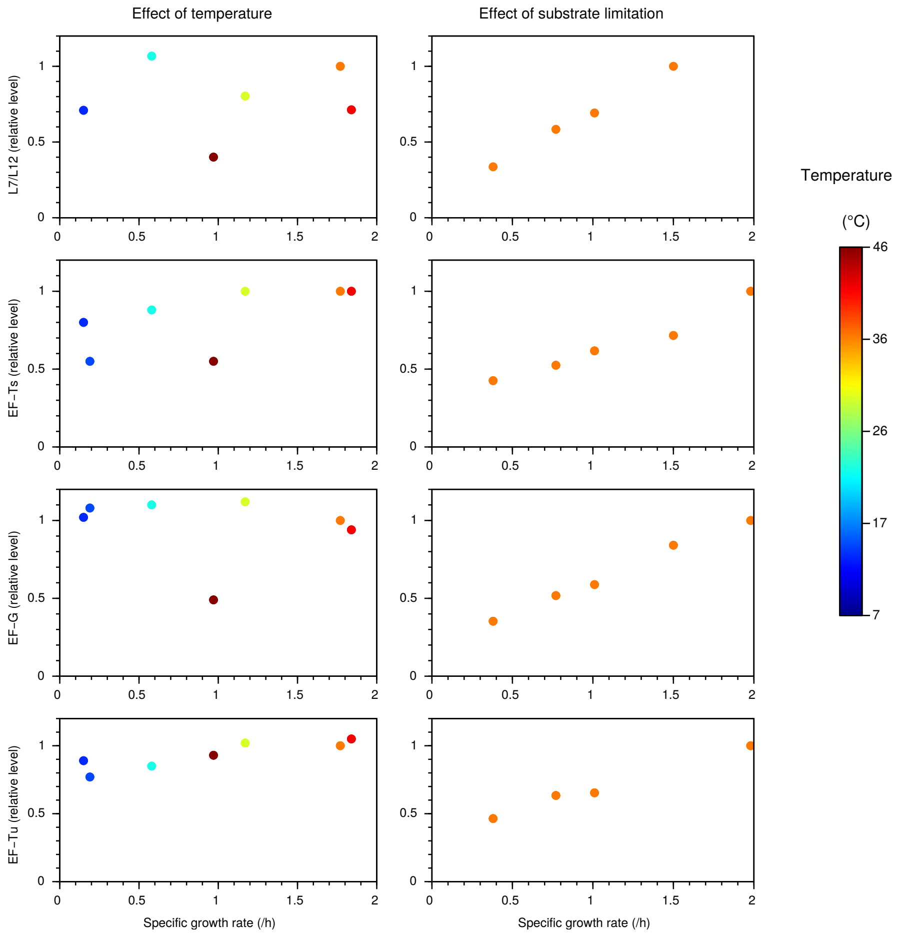

Supplementary Figure 1: Protein content for the ribosomal sector in *E. coli* as a function of specific growth rate, varying with temperature (left) or substrate limitation (right). Level of the proteins L7/L12, EF-Ts, EF-G, and EF-Tu (from top to bottom) for different temperatures in glucose rich medium (left), and for different media at 37 °C, relative to glucose-rich medium at 37 °C [3, 4]. For each point, color represents temperature (see colorbar). The predicted resource allocation profiles plotted in Fig. 3 correspond remarkably well with the experimental data shown here.

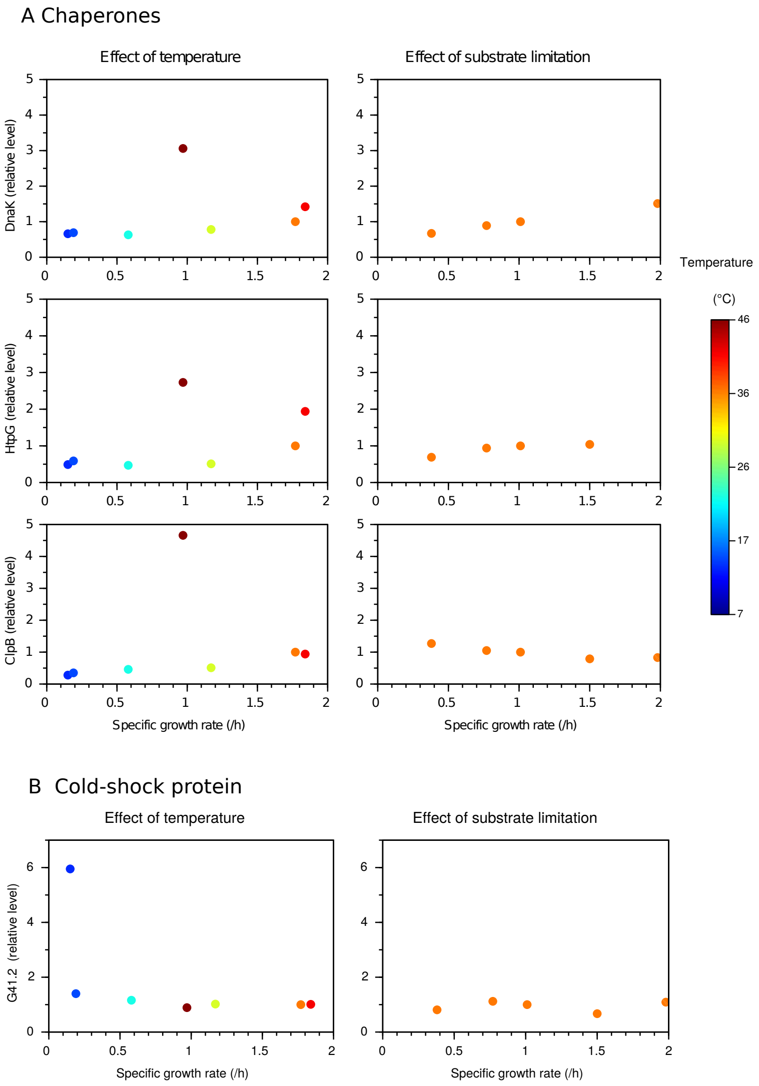

Supplementary Figure 2: Chaperone and cold-shock protein contents in *E. coli* as a function of specific growth rate, varying with temperature (left) or substrate limitation (right). Same legend as Supplementary Figure 1. (A) Chaperones DnaK, HtpG, and ClpB from top to down. (B) Cold-shock protein G41.2. Contrary to the model predictions, the chaperone content in (A) increases only at high, but not at low temperatures. Actually, different proteins are involved for cold and heat stresses. Cold-induced proteins have been observed in [3] and [4] (*e.g.*, protein G41.2 shown in (B)), but their functions remain unknown or do not correspond to chaperones. Since these studies, other cold-induced proteins have been identified with a role of chaperone for RNA/DNA or proteins [5]. The global trend of the chaperone sector is thus in line with the model predictions, when aggregating both cold and heat-shock chaperones.

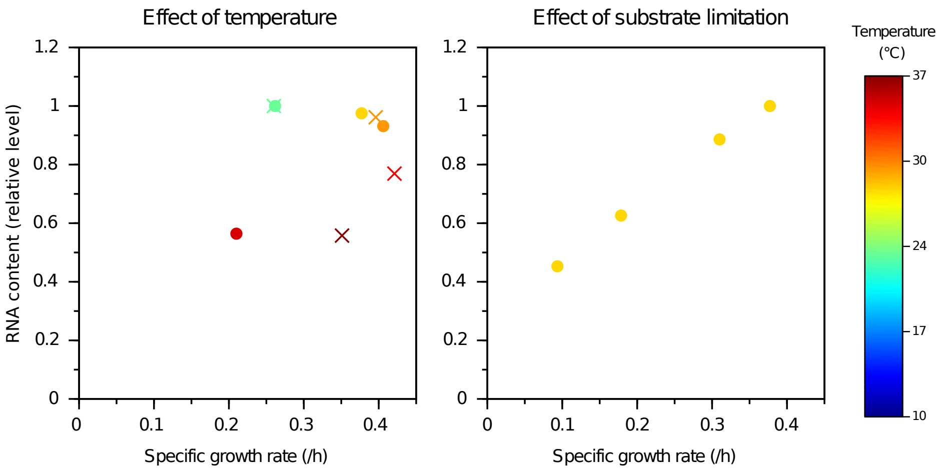

Supplementary Figure 3: RNA content in *S. cerevisiae* as a function of specific growth rate, varying with temperature (left) or substrate limitation (right). Data from [6] (cross) in batch culture, and from [7] (circle) in batch (left) and chemostat (right). For each symbol, color represents temperature (see colorbar). *S. cerevisiae* shows very similar trends to *E. coli* and model predictions (see Fig. 3).

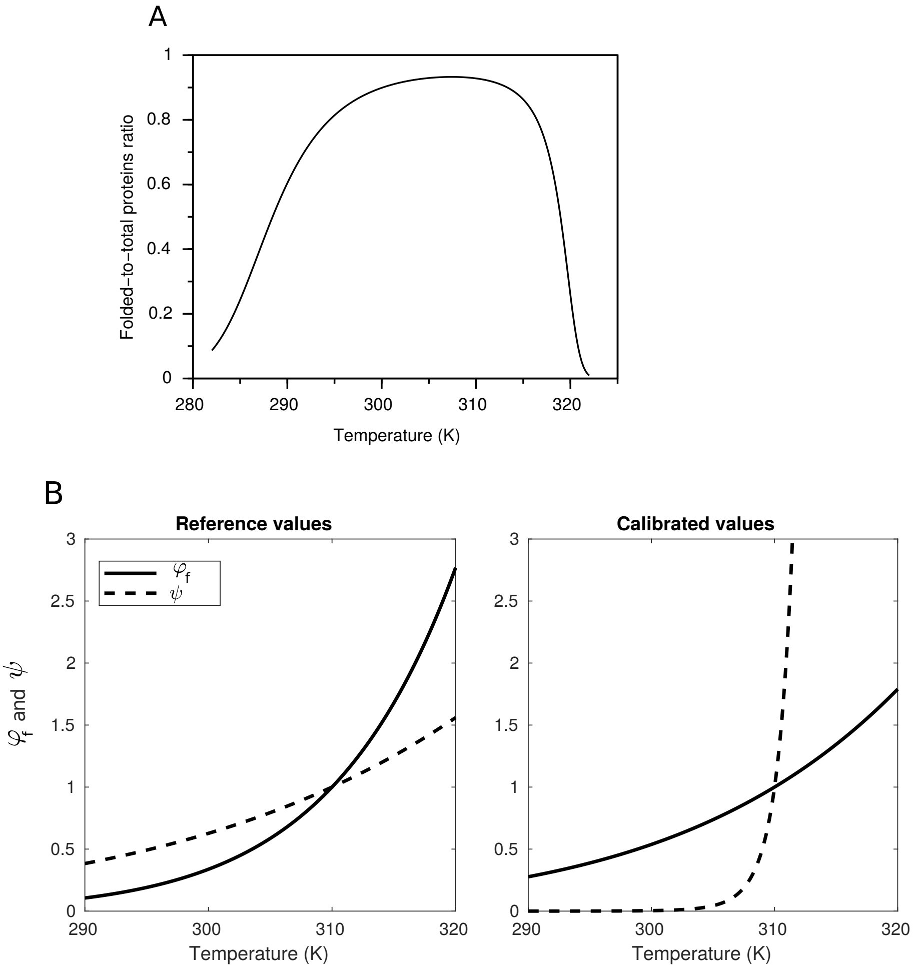

Supplementary Figure 4: (A) The predicted ratio of folded-to-total proteins η as a function of temperature (see Eq. (8) of the main article). (B) Functions $\varphi_f(T)$ and $\psi(T)$ using for parameters the reference values (left) or the calibrated values (right).

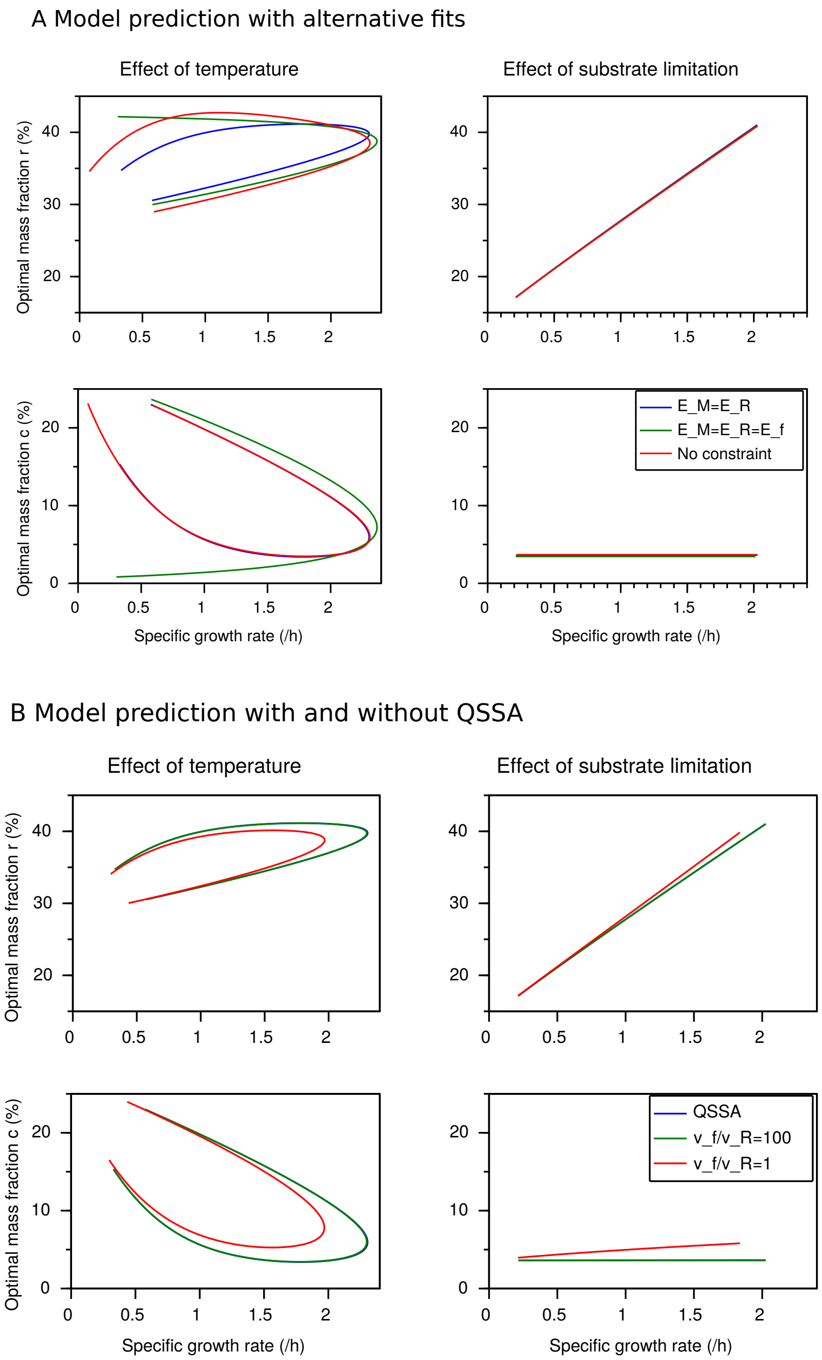

Supplementary Figure 5: Robustness of model predictions. (A) Comparison of the optimal solutions for alternative fits. The trends remain similar, except the fit with $E_M = E_R = E_f$ (which does not represent chaperone synthesis at low temperatures). B) Comparison of the optimal solutions of the QSSA and the full models, for two different values of the folding rates. The blue and green curves are superposed, demonstrating that the QSSA gives a good approximation of the full model.

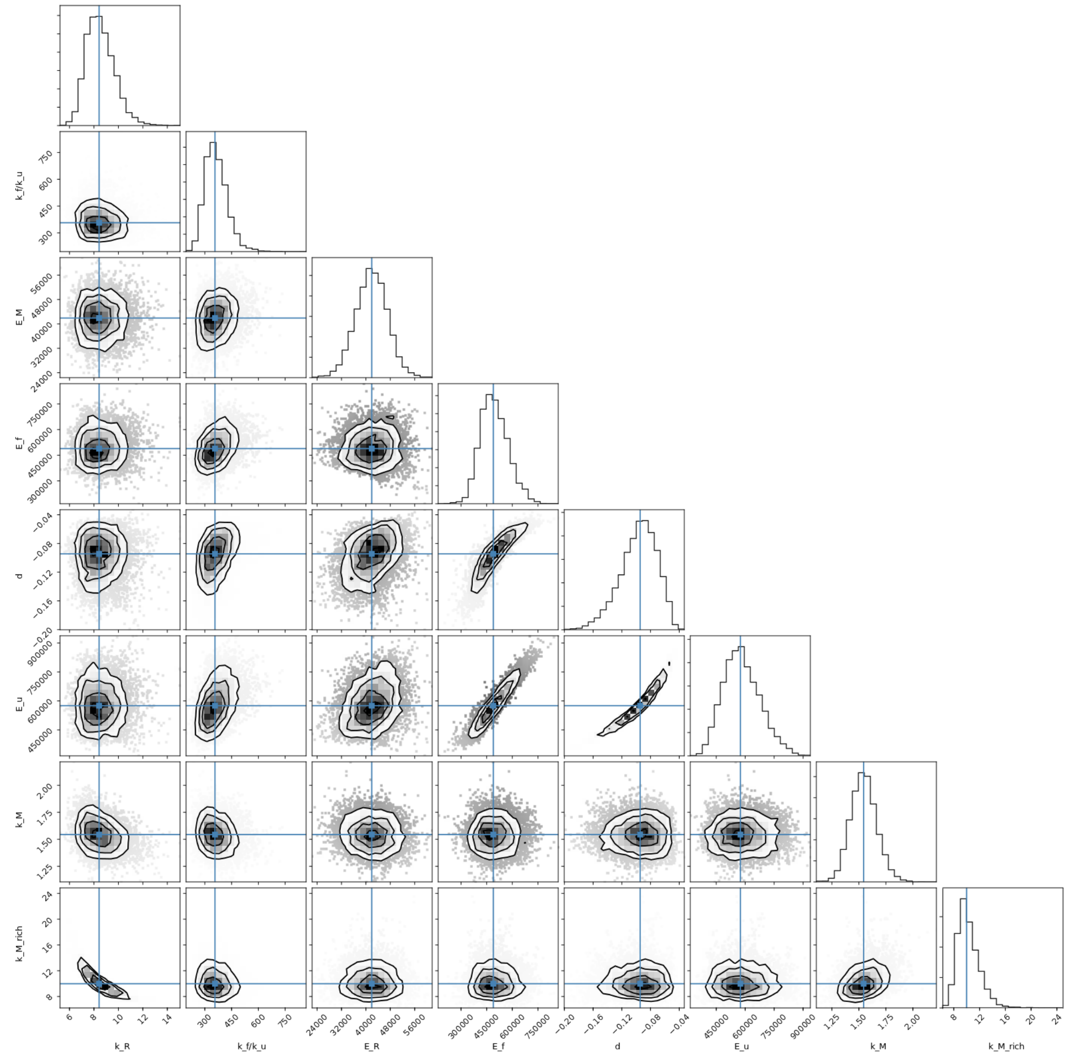

Supplementary Figure 6: Pairwise posterior distribution of model parameters, obtained with a Monte-Carlo Markov Chain algorithm. See Methods 4.2 in the main text for details of the computation. The three parameters representing the effect of temperature on protein folding and unfolding (E_f, E_u and d) are correlated.

References

[1] Dill KA, Ghosh K, Schmit JD. Physical limits of cells and proteomes. Proc Natl Acad Sci U S A. 2011;108(44):17876–17882.

[2] Heinrich R, Schuster S. The Regulation of Cellular Systems. Springer Science & Business Media; 2012.

[3] Herendeen SL, Vanbogelen RA, Neidhardt FC. Levels of major proteins of *Escherichia coli* during growth at different temperatures. J Bacteriol. 1979;139(1):185–194.

[4] Pedersen S, Bloch PL, Reeh S, Neidhardt FC. Patterns of protein synthesis in *E. coli*: a catalog of the amount of 140 individual proteins at different growth rates. Cell. 1978;14(1):179–190.

[5] Barria C, Malecki M, Arraiano C. Bacterial adaptation to cold. Microbiology. 2013;159(12):2437–2443.

[6] Benet M, Miguel A, Carrasco F, Li T, Planells J, Alepuz P, et al. Modulation of protein synthesis and degradation maintains proteostasis during yeast growth at different temperatures. Biochim Biophys Acta, Gene Regul Mech. 2017;1860(7):794–802.

[7] Parada G, Acevedo F. On the relation of temperature and RNA content to the specific growth rate in *Saccharomyces cerevisiae*. Biotechnol Bioeng. 1983;25(11):2785–2788.
